# Molecular Pathways, Target Landscape, and Translational Models in Heart Failure with Preserved Ejection Fraction

**DOI:** 10.3390/cells15171588

**Published:** 2026-09-01

**Authors:** Haleema Qamar, Janmeet S. Saini, Sylwia Marta Figarska, Emmanuel S. Buys, Vilas Wagh

**Affiliations:** 1 Data AI and Genome Sciences (DAGS), Merck & Co., Inc., Cambridge, MA 02141, USA; 2 Cardiometabolic Disease (CMD), Merck & Co., Inc., South San Francisco, CA 94080, USA

**Keywords:** heart failure, heart failure with preserved ejection fraction (HFpEF), cardiometabolic diseases, cardiac precision medicine, cardiovascular diseases, phenotypic heterogeneity

## Abstract

**Highlights:**

**What are the main findings?**

- HFpEF’s clinical heterogeneity is attributable to underlying interconnected molecular pathways that drive the disease, and the therapeutic landscape is evolving toward phenotype-specific interventions.
- Current animals and in vitro HFpEF fail to fully recapitulate the mechanistic complexity of the disease spectrum, and thus there is a major need for improving phenotype dependent translational fidelity.

**What are the implications of the main findings?**

- Advancing the effectiveness of HFpEF will likely rely on improved phenotype-based patient stratification strategies, which remains a key area of investigation.
- Innovation in drug discovery and clinical translation requires prioritization of human-relevant, multifactorial HFpEF models with integration of high-throughput functional assays, multi-omics profiling, and machine-learning approaches to improve patient-specific therapeutic outcomes.

**Abstract:**

Heart failure with preserved ejection fraction (HFpEF) is a substantial global health burden and the greatest unmet medical need for cardiovascular diseases. It is marked by pronounced clinical heterogeneity and complex multi-system pathophysiology with limited therapeutic options. Progress in developing effective therapeutics is constrained by the inadequacy of experimental models to fully recapitulate the multifactorial nature of the disease. Recent evidence underscores the significant involvement of inflammatory, oxidative, and mitochondrial pathways in the pathogenesis of HFpEF, with non-coding RNAs and epigenetic regulation serving as crucial modulators and prospective therapeutic targets. This review maps the HFpEF target landscape, while critically assessing the mechanistic contributions, translational fidelity, and limitations of existing in vivo and in vitro models. Further, advances are noted among the in vitro technologies, including human cardiac organoids and engineered heart tissues integrated with high-throughput multi-omics and computational modeling, enabling in-depth examination of HFpEF mechanisms. Finally, we underscore the necessity of integrative, systems-level approaches and multi-marker strategies to enhance translational relevance, improve risk stratification, and accelerate development of mechanism-based therapies. Collectively, this review supports phenotypic-guided and mechanism-informed therapeutic development for HFpEF, and provides a roadmap for next generation model development and therapeutic innovation.

## 1. Introduction

**Figure 1:**
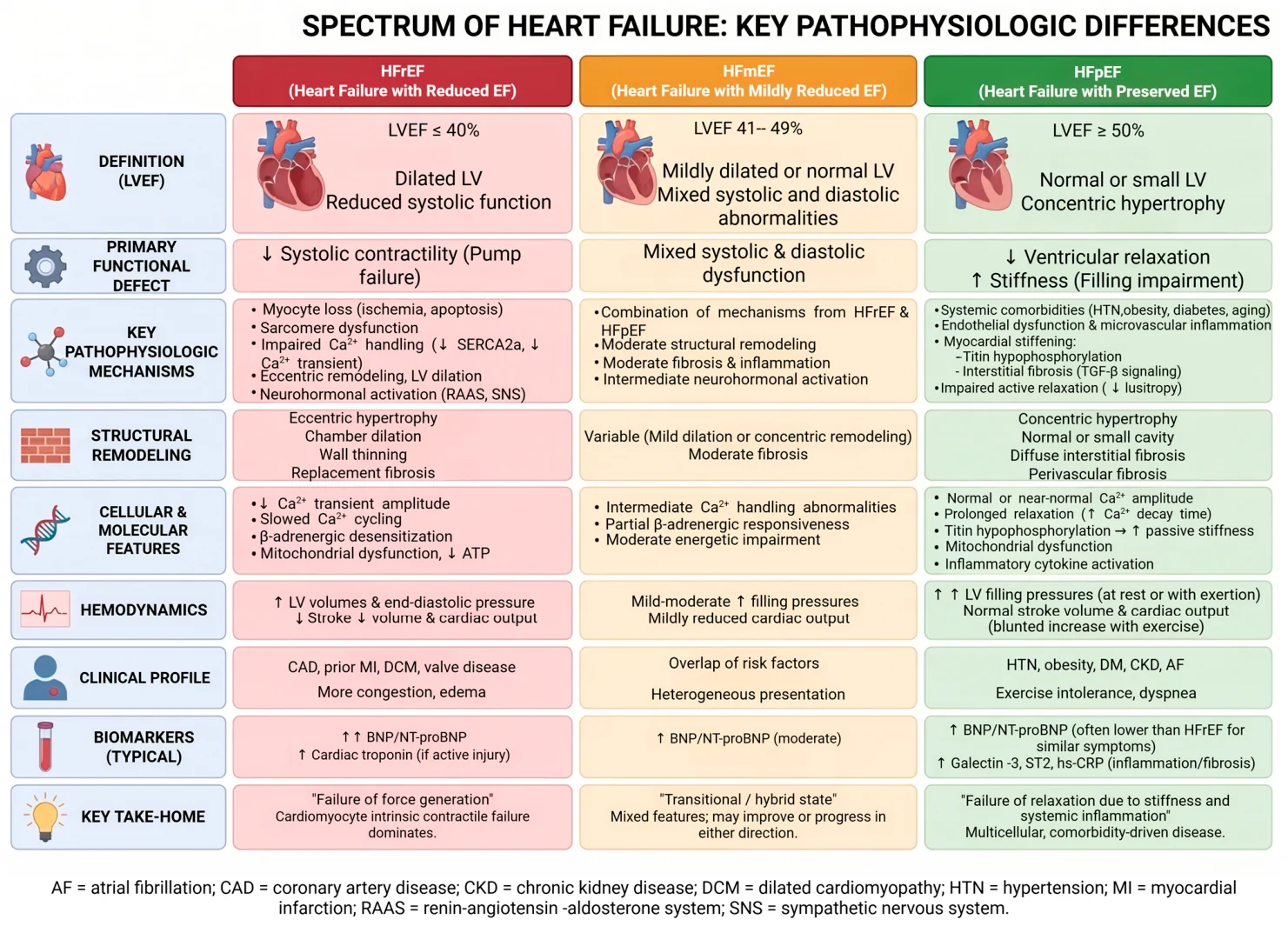
Characterization of heart failure based on the ejection fractions. Fundamental functional and mechanical differences among three types of heart failure. (↓ = decrease, ↑ = increase).

Heart failure (HF) is characterized by structural or functional cardiac impairment that disrupts ventricular filling or ejection, limiting the cardiac ability to pump blood, resulting in failure to meet overall metabolic demands. HF is categorized based on left ventricular ejection fraction (LVEF), and three major subtypes include HF with reduced ejection fraction (HFrEF, LVEF ≤ 40%), mildly reduced ejection fraction (HFmEF, LVEF = 41–49%), and preserved ejection fraction (HFpEF, LVEF ≥ 50%). Besides these numerical variations in cardiac output, while there is overlap in disease-driving mechanisms, HFpEF and HFrEF have certain unique pathophysiological molecular pathways that define response to therapeutic interventions. Figure 1 captures the nuances among the three HF subtypes in functional and structural abnormalities and details of mechanical distinctions. Further highlighting these mechanistic distinctions are the conventional therapies that are effective for HFrEF but have proven ineffective against HFpEF [1].

HFpEF, also known as diastolic heart failure, includes more than half of the total HF cases and is a growing challenge due to its increasing prevalence, complex pathophysiology, high morbidity, and mortality [2]. HFpEF is characterized by a constellation of cardiac and extracardiac abnormalities, including diastolic dysfunction, myocardial fibrosis, systemic inflammation, and metabolic dysregulation, which collectively contribute to exercise intolerance, poor prognosis, and high morbidity [3]. Despite its clinical significance, developing effective therapies for HFpEF has been challenging. One of the reasons is the failure of experimental models to faithfully recapitulate the complex multifactorial nature of the disease. Absence of validated molecular targets for intervention adds another layer of complexity in advancing therapeutic progress [4]. The translation of preclinical findings to clinical benefit stays elusive, attributable to simplistic study designs. The heterogeneity of HFpEF, with its diverse comorbidities and systemic manifestations, complicates both diagnosis and management and thus underscores the need for innovative disease modeling systems and more precise molecular characterization [5].

Traditional HFpEF animal models mostly focused on hemodynamic abnormalities and pressure overload or induced the disease phenotype by focusing on a single comorbidity, such as hypertension or diabetes. These drawbacks limit the utility of current in vivo experimental models for preclinical evaluation of new therapeutic targets. However, the contemporary paradigm shift that presents HFpEF as a complex multisystemic syndrome has led to experimental designs incorporating multifactorial disease drivers to improve model fidelity. The screening methodologies for evaluation of molecular targets are also being diversified to find underlying pathways involved in inflammation, oxidative stress, mitochondrial dysfunction, and impaired autophagy [6]. Despite recent developments, biomarker-based diagnostic strategies for HFpEF remain inadequate for precision diagnosis and personalized disease management. This gap reflects the syndrome’s substantial comorbidity burden, analytical variability across biomarker platforms, and the lack of robust multi-marker diagnostic panels [7]. These challenges reinforce the need for disease models and therapeutic targets that capture the clinical complexity and dynamic pathophysiology of the disease [8]. This study integrates the evidence that supports HFpEF as a complex multiorgan syndrome with clinically distinct phenotypes and follows the therapeutic landscape evolution from proven to emerging targets. It recognizes the progress made with human cardiac organoids, engineered heart tissue, and multi-omics data integration to dissect the molecular and cellular mechanisms underlying HFpEF while also critically assessing the strengths and limitations of current experimental models along with highlighting the future roadmap [9]. This narrative review synthesizes pertinent clinical, translational, and mechanistic literature concerning HFpEF molecular pathways, treatment targets, biomarkers, and experimental models. This is not designed as a systematic review or meta-analysis; thus, assertions are presented as a critical synthesis rather than a systematic mapping of the domain.

## 2. HFpEF Pathophysiology and Target Landscape

The exhaustive understanding of HFpEF requires focus on its molecular, cellular, and systemic targets, as well as the evolving models and biomarkers that inform therapeutic strategies. Historically, “HFpEF” was simply seen as dysfunction of diastolic, characterized by myocardial hypertrophy and stiffness, resulting in impaired relaxation kinetics and elevated filling pressures. Extensive phenotyping and data-driven clustering studies have found multiple HFpEF phenotypes based on a distinct set of comorbidities, demographics, hemodynamics, and biomarker profiling. The heterogeneity in clinical manifestation is dictated by the associated comorbidity burden, such as hypertension, obesity, type 2 diabetes (T2D), chronic kidney disease (CKD), chronic obstructive pulmonary disease (COPD), and aging [3].

Over the past decade, mechanistic studies have reframed HFpEF as a complex multiorgan syndrome involving diverse cardiac and extracardiac abnormalities that affect not only myocardial structure and function but also the vasculature, kidneys, lungs, and skeletal muscle. Comorbidity-driven pathways promote systemic inflammation, cardiac fibrosis, endothelial activation, abnormal energetics, and coronary microvascular dysfunction, collectively contributing to myocardial stiffening and diastolic dysfunction in HFpEF [10]. We dissect the underlying pathophysiological processes, pathways involved, and therapeutic target analysis within those pathways. These pathways should be viewed as overlapping disease mechanisms and candidates for therapeutic targets, not as independently validated treatment axes.

## 3. Coronary Microvascular and Endothelial Dysfunction

In 2013, Paulus and Tschöpe introduced a microvascular-inflammation paradigm that centralizes chronic low-grade inflammation, driven by the various comorbidities, as a cardinal feature of the HFpEF pathophysiological framework [11]. This proinflammatory state induces microvascular endothelial inflammation, marked by upregulation of adhesion molecules such as VCAM-1, ICAM-1, and E-selectin in myocardial microvascular endothelial cells. Activated endothelium recruits immune cells and promotes reactive oxygen species (ROS) generation, which reduces nitric oxide (NO) bioavailability and disrupts soluble guanylate cyclase (sGC) signaling. Impaired NO–sGC signaling lowers cyclic GMP (cGMP) levels and suppresses protein kinase G (PKG) activity in adjacent cardiomyocytes. The ultimate downstream consequence is titin hypo phosphorylation at its N2B unique sequence and PEVK domain that increases cardiomyocyte passive stiffness and disrupts ventricular relaxation kinetics [12].

This framework is clinically supported by HFpEF patients with higher levels of circulating C-reactive protein (CRP), TNF-α, IL-1β, IL-6, and GDF-15, correlating with exacerbating diastolic dysfunction. The burden of comorbid conditions highly correlates with CRP levels, thus confirming comorbidities as an upstream source of inflammation [13,14].

Inflamed microvascular endothelium expresses ICAM and VCAM, becomes permeable for immune cell infiltration into the myocardium, and triggers the NLRP3 inflammasome and release of IL-β1 and IL-18, reinforcing local inflammation. Macrophages also excrete TGFβ and other profibrotic factors, stimulating fibroblast activation, ECM remodeling, and fibrosis. Chronic inflammation can induce cardiomyocyte hypertrophy, titin modifications, altered calcium handling, metabolic shift, and endothelial–myocyte uncoupling [14]. Inflammation also underlies skeletal muscle abnormalities and cachexia correlated with HFpEF [15]. Microvascular dysfunction eventually leads to capillary rarefaction and impaired myocardial blood flow that gives rise to the hallmark hemodynamic abnormalities. The physiological result is diminished exercise capacity (peak VO_2_) due to the heart and skeletal muscles’ inability to adequately increase blood flow under stress [16,17].

While an inflammatory state is fueled by multiple accompanying indications, metabolic diseases such as obesity and T2D are specifically impactful in creating a proinflammatory state through the secretion of proinflammatory adipokines. Similarly, aging is characterized by inflammaging, a state of chronic inflammation with elevated cytokines, and immune activation [18]. Patients with an “aging phenotype” of HFpEF have particularly higher rates of hospitalization and mortality [19].

Aging causes arterial stiffening, evident by widened pulse pressures and higher central pressures in older HFpEF patients. The vascular stiffening promotes left ventricular remodeling, left atrial enlargement, and disrupted ventricular relaxation kinetics. Aging also leads to senescence-related systemic inflammation, nitrosative stress, oxidative stress, and endothelial dysfunction, triggering fibroblast activation and matrix stiffening [20]. Older HFpEF patients often have sarcopenia and frailty, further reducing exercise capacity [18,21,22].

## 4. Cardiometabolic Stress and Mitochondrial Impairment

Many HFpEF patients can be categorized as having the “cardiometabolic” HFpEF phenotype driven by metabolic syndrome, obesity, and insulin resistance in addition to dyslipidemia, sedentary lifestyle, and obstructive sleep apnea. This axis involves loss of metabolic flexibility, mitochondrial dysfunction, and energy deficits underlined by cellular inability to efficiently switch between fuel sources (glucose, fatty acids, and ketones), fostering lipotoxicity (steatosis), along with systemic metabolic inflammation. These conditions impose chronic nutrient overload and hyperinsulinemia, promoting insulin resistance in skeletal muscles and likely in cardiomyocytes. Elevated circulating free fatty acids (FFA) and hyperglycemia over time drive lipotoxicity and glucotoxicity in tissues [23].

Healthy hearts oxidize FFAs predominantly but can switch to glucose metabolism in stress or ischemia [15]. The metabolic flexibility in HFpEF is either blunted by an increase in fatty acid oxidation and drop in glucose use, or sometimes the fatty acid oxidation is impaired altogether along with glucose metabolism. Both scenarios result in inefficient ATP production and accumulation of toxic lipid intermediates, triglycerides, and ROS content, provoking maladaptive signaling. At the cellular level, less ATP for SERCA2a function can potentially slow the calcium reuptake and contribute to diastolic dysfunction. Metabolic dysregulation is central to mitochondrial impairment, with obesity, diabetes, and hypertension promoting the expansion of visceral and epicardial adipose tissue, which secrete proinflammatory adipokines (e.g., leptin, TNF-a, and IL-6) and profibrotic mediators, leading to increased myocardial stiffness and diastolic dysfunction [24].

## 5. Myocardial Fibrosis and ECM Remodeling

The interstitial and perivascular fibrosis driven by activated myofibroblasts, TGF-β signaling, galectin-3, soluble ST2, collagen crosslinking, and TIMP-1 is the central contributor to the elevated passive left ventricular stiffness in HFpEF. The multi-omics characterization of LV biopsies from cardiometabolic HFpEF patients shows marked ECM remodeling (enrichment of fibrillar collagen proteins, fibronectin, and POSTN) correlating with indices of diastolic dysfunction and fibrosis, occurring independently of obesity [25]. TGF-β signaling programs, NF-κB activation, and myofibroblast–macrophage coactivation form a self-reinforcing fibrotic loop [26].

Fibrosis in HFpEF is driven by multiple comorbidities. Chronic hypertension induces pressure overload and activates neurohormonal profibrotic pathways, including RAAS and TGF-β signaling. Chronic kidney disease further exacerbates RAAS activation. Aging and systemic inflammation create a pro-fibrotic immune environment, where activated immune cells sustain myofibroblast phenotypes and promote the deposition of collagen types I and III, ultimately increasing tissue stiffness [27].

TGF-β1 is the key molecular trigger that drives fibroblast activation via SMAD signaling. Angiotensin II and aldosterone, mediators of the hypertensive phenotype, can upregulate TGF-β production and enhance fibroblast proliferation. Matrix crosslinking and stiffness further worsen with endothelin-1 from endothelial cells and galectin-3, a macrophage-derived lectin, promoting fibroblast collagen and fibronectin production. HFpEF hearts may show a shift toward the stiffer N2B titin isoform compared to the more compliant N2BA isoform, although this contribution is generally modest compared with alterations in titin phosphorylation. Additionally, mechanosensitive pathways in cardiomyocytes (like FAK and integrin signaling) may also propagate fibrotic remodeling in response to increased stiffness. Fibrosis, in HFpEF, can further induce atrial fibrosis and possibly ventricular arrhythmia [28,29].

## 6. Cardio-Renal HFpEF

HFpEF often co-exists with CKD, and impaired renal function is both a risk factor and a consequence of HFpEF. This reflects the bidirectional cardiorenal syndrome where chronic elevation of left ventricular filling pressures causes renal venous congestion, reduced renal perfusion, and neurohormonal activation. The hemodynamic changes, coupled with underlying CKD, trigger renin–angiotensin–aldosterone system (RAAS) and sympathetic nervous system (SNS) activation that helps water retention, which cycles back to contributing to elevated filling pressures in HF. Similarly, CKD promotes endothelial dysfunction, accumulation of uremic toxins, and systemic inflammation resulting in myocardial fibrosis and stiffness. On a cellular level, angiotensin II and aldosterone trigger myocardial hypertrophy and, as noted earlier, stimulate fibrosis [30].

## 7. Ventricular-Vascular Coupling and Stiffening in HFpEF

Ventricular and vascular stiffness play a leading role in HFpEF biology, primarily through disruption of ventricular–arterial coupling, the balance between left ventricular function and arterial load. Arterial and ventricular stiffening raises cardiac filling pressures and reduces left ventricular compliance, limiting the cardiac ability to increase output during exertion. This pathway highlights HFpEF as a disease of both the heart and the circulation [14].

Chronic pressure and oxidative stress in arteries induce hypertrophy in vascular smooth muscle cells (VSMCs) and trigger VSMCs’ transition into a pro-inflammatory, osteoblastic phenotype, causing calcification in arterial media. Endothelial dysfunction from risk factors impairs vasodilatory capacity and increases systemic vascular resistance. In response, the ventricle becomes hypertrophied and ECM stiffens, along with alteration in titin isoform expression (N2B:N2A). Impaired ventricular–arterial coupling causes an exaggerated blood pressure response during exertion because of arterial stiffness and limits stroke volume augmentation because of a stiff, low-reserve left ventricle. Thus, HFpEF patients cannot adequately increase stroke volume with exercise, relying solely on heart rate increase, which is limited by chronotropic incompetence. Thus, an early plateau of cardiac output is exhibited along with early fatigue [16,31].

## 8. Pulmonary Vascular Remodeling in HFpEF

A subset of HFpEF patients develops pulmonary hypertension (PH) and right ventricular dysfunction. Chronically elevated left ventricular pressures and pulmonary vascular remodeling can lead to severe postcapillary PH. Over time, this triggers pulmonary arterial remodeling and leads to right ventricle dysfunction. The right ventricle goes through hypertrophy and eventually dilates as it struggles with adapting to increased pulmonary pressures, leading to tricuspid regurgitation and systemic congestion [32].

## 9. Epigenetic Regulation and Non-Coding RNA in HFpEF

Compounding research highlights the role of non-coding RNAs and epigenetic modifications in HFpEF pathogenesis and potential therapy. MicroRNAs (miRNAs), long non-coding RNAs (lncRNAs), and circular RNAs (circRNAs) are vital regulators of gene expression and have been implicated in cardiac remodeling, fibrosis, hypertrophy, and inflammation [33,34].

MicroRNAs (miRNAs): Numerous miRNAs have been found to be associated with HFpEF phenotypes; however, their diagnostic and therapeutic utility is still investigational because reproducibility, tissue specificity, assay harmonization, and external validation remain unresolved. For instance, miR-21 is a known regulator of cardiac fibrosis and is elevated in the plasma of obese individuals with diastolic dysfunction and in some HFpEF patients [35]. Another example is miR-181c, which is elevated in patients with diabetic HFpEF phenotype and blunts exercise response by promoting fibrosis by targeting SMAD7 and PRKN [36]. A seven-miRNA circulating panel, including let-7a-5p, miR-125a-5p, and miR-150-5p, has been reported to distinguish HFpEF from HFrEF, highlighting the potential of miRNAs as a HFpEF biomarker signature [37]. Additionally, gender-based expression variations were seen in women with diabetes and HFpEF, correlating with microvascular damage. These findings depict miRNAs both as mechanistic contributors and as prognostic biomarkers in HFpEF [38].

Long non-coding RNAs (lncRNAs) such as LIPCAR, MIAT, and MHRT have been found upregulated in samples from HFpEF patients, and some are associated with severity of diastolic dysfunction. Another lncRNA, MALAT1, drives cardiac pathology induced by diabetes, a comorbidity in HFpEF, and correlates with endothelial cell dysfunction and reduced capillary density [33,39].

Circular RNAs (circRNAs) are covalently closed, stable RNA molecules that can regulate gene expression and function as miRNA sponges. Some circRNAs (e.g., circHIPK3) are downregulated in cardiac hypertrophy and fibrosis models, but their roles in HFpEF remain under active investigation. Through interactions with miRNAs or RNA-binding proteins, circRNAs may influence signaling pathways involved in fibrosis, such as TGF-β/SMAD, and hypertrophy, including PI3K-AKT. Overall, these fine regulators can influence the gene expression landscape in HFpEF, affecting hypertrophy, fibrosis, and metabolism, among others [33,34].

Beyond non-coding RNAs, epigenetic modifications may also contribute to HFpEF. DNA methylation patterns and histone modifications have been linked to pathological hypertrophy and fibrosis in HFpEF. For example, Class I HDACs are upregulated in myocardial samples from patients with HFpEF, and their inhibition improves cardiac fibrosis and diastolic function in preclinical models [40]. BET (bromodomain and extra-terminal) proteins, which promote pro-inflammatory gene transcription, have also been proposed as therapeutic targets for reducing HFpEF-associated inflammation and fibrosis [41].

## 10. Sex-Specific Mechanisms

**Figure 2:**
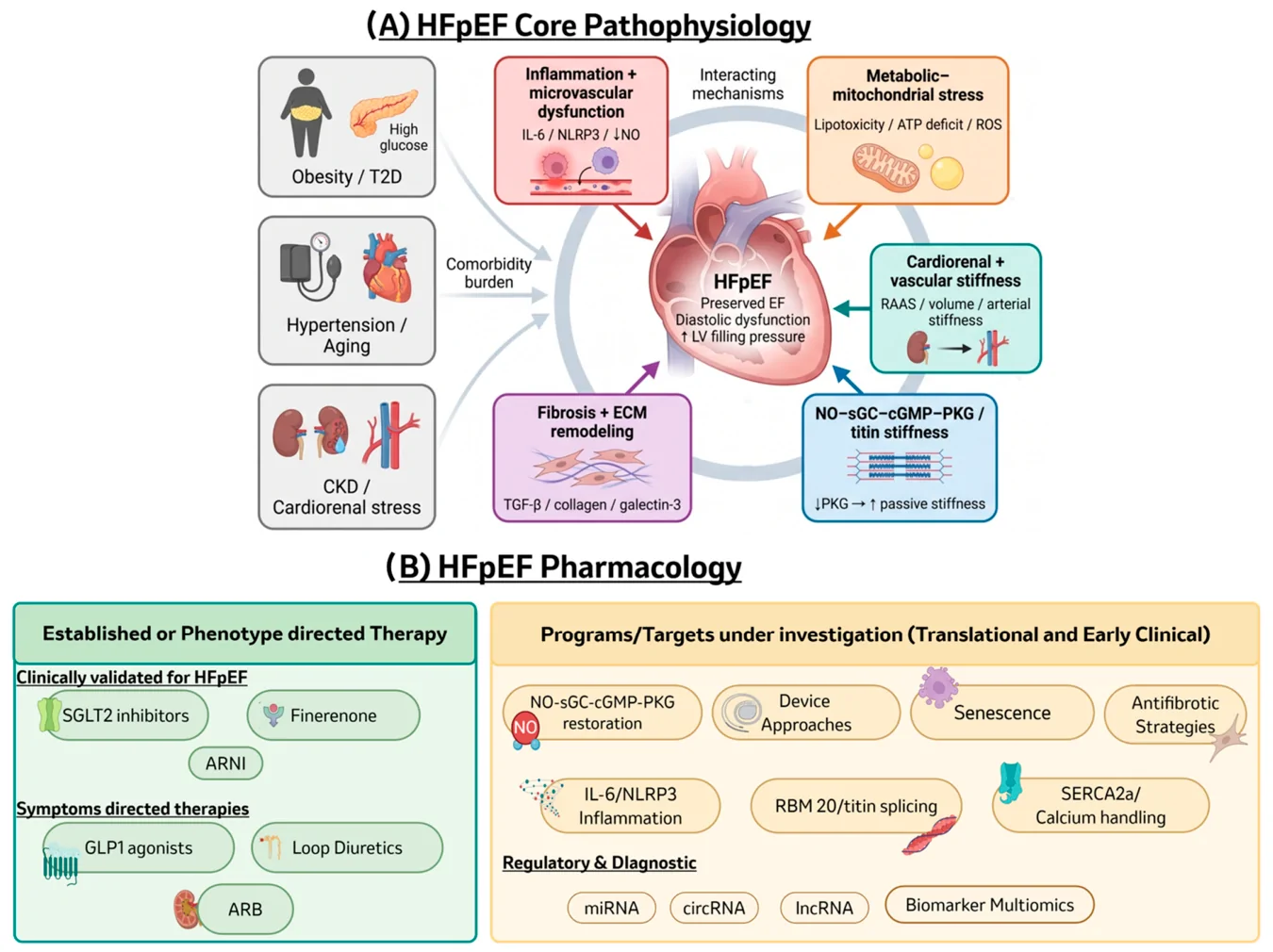
Pathophysiologic mechanisms and therapeutic landscape of heart failure with preserved ejection fraction (HFpEF). (**A**) Schematic overview of the multifactorial pathogenesis of HFpEF. Common cardiometabolic and age-related comorbidities, including obesity/type 2 diabetes (T2D), hypertension, aging, and chronic kidney disease (CKD), impose a cumulative systemic burden that promotes interconnected pathological processes. The convergence of pathological mechanisms leads to preserved left ventricular ejection fraction (LVEF) despite impaired diastolic relaxation, elevated left ventricular filling pressures, reduced cardiac reserve, and exercise intolerance, which collectively define the HFpEF phenotype. (**B**) Current and emerging therapeutic strategies for HFpEF. Established or clinically validated approaches include sodium-glucose cotransporter-2 (SGLT2) inhibitors, finerenone and ARNI, while incretin-based therapies, diuretics and antihypertensive agents are effective for managing underlying complications. Other areas of active development include senescence-targeted therapies and molecular diagnostics using non-coding RNAs (miRNAs, circRNAs, lncRNAs) and multi-omics biomarkers for improved patient stratification. (↓ = decrease, ↑ = increase).

HFpEF predominantly affects women more than men, in contrast to HFrEF, which is more common in men. Women with HFpEF tend to be in older age with a distinct remodeling pattern of disease that reflects sex hormones and related biological changes. Additionally, the disease manifests with more severe hypertension, smaller left ventricular cavity size, and greater concentric remodeling in women than in men [38,42]. In post-menopausal changes, loss of estrogen’s cardioprotective effects may worsen microvascular dysfunction and promote fibrosis in women with HFpEF. Emerging evidence also shows sex-specific molecular differences. For example, variations in microRNA signatures in women with HFpEF and diabetes correlate with a higher incidence of microvascular injury and diastolic dysfunction. These differences suggest that gender may influence the dominant HFpEF pathways in a way that women might be more likely to exhibit microvascular inflammation and hypertensive remodeling, while men may show greater involvement of ischemic or microvascular mechanisms. These differences can also dictate response to the drug therapies, e.g., the PARAGON-HF trial showed that women might derive a greater benefit from ARNI therapy than men, possibly due to differences in signaling pathways [43]. Figure 2 provides a general snapshot of the HFpEF target landscape.

## 11. Interventions Against HFpEF

Key signaling pathways and biological processes relevant to HFpEF include the inflammatory response, vasculature development, regulation of the MAPK cascade, apoptotic signaling, response to oxidative stress, and regulation of cell adhesion [44]. At the gene level, differentially expressed genes, tissue-specific hub genes, and shared hub genes have been identified as potential therapeutic targets [45]. Non-coding RNAs, including microRNAs, long non-coding RNAs, and circular RNAs, are increasingly recognized as regulators of HFpEF pathophysiology and offer a novel class of therapeutic targets.

Obesity and visceral adiposity are central mechanisms linking inflammation and immune dysregulation to HFpEF, making them important therapeutic targets. GLP-1 receptor agonists and weight-loss drugs are promising strategies for targeting obesity in HFpEF [24]. The main therapeutic targets in HFpEF include diastolic dysfunction and elevated left ventricular filling pressure, as these are central features of the syndrome [44].

## 12. HFpEF Target Landscape

At present, therapeutic evidence in HFpEF is not uniform across targets. SGLT2 inhibitors are the most consistently validated pharmacological class for reducing worsening heart failure events across the preserved and mildly reduced ejection fraction spectrum. Finerenone has recently shown favorable outcome data in patients with LVEF ≥ 40%, while incretin-based therapies currently appear most relevant to obesity-related HFpEF phenotypes. Most anti-inflammatory, antifibrotic, epigenetic, and regenerative approaches are still under investigation.

### 12.1. Evidence-Based and Clinically Actionable Therapeutics Used in HFpEF

This section comprises interventions supported by large, randomized trials and current regulatory approval, such as SGLT2 inhibitors and finerenone for HF with LVEF ≥ 40% where approved, along with complementary drug strategies to manage underlying comorbidities, such as GLP-1 agonists and anti-hypertension drugs, providing symptomatic relief.

#### 12.1.1. Approved HFpEF Pharmacotherapies

##### Sodium-Glucose Cotransporter-2 Inhibitor (SGLT2i)

Sodium-glucose cotransporter 2 inhibitors (SGLT2is) act beneficially across the spectrum of left ventricular ejection fraction, addressing the unmet clinical need for HFpEF treatment. SGLT2i compounds, notably empagliflozin, dapagliflozin, and sotagliflozin, are first-class drugs with wide guideline recommendations across the HF ejection fraction spectrum and the current cornerstone of HFpEF pharmacotherapy. Empagliflozin reduced the composite of CV-worsening events and heart failure hospitalization in the EMPEROR-Preserved trial, with the benefit driven primarily by a reduction in heart failure hospitalization. Similarly, the DELIVER trial confirmed equivalent benefits with the dapagliflozin. They have become first-line therapy in HFpEF because they address multiple metabolic and hemodynamic derangements at once [46].

The efficacy of SGLT2i in HFpEF proves the relevance of metabolic pathways in disease pathophysiology. SGLT2i mechanisms in HFpEF are pleiotropic and extend well beyond glycosuria. They cause osmotic diuresis (reducing volume), direct suppression of myocardial inflammation via inhibition of ICAM-1, VCAM-1, IL-6, and TNF-α. Additionally, they also play a role in restoration of NO-sGC-cGMP-PKG signaling and reduction of titin-based passive stiffness through improved cardiomyocyte cGMP, while offering weight loss benefits by reducing epicardial adipose tissue and enhancement of ketone body oxidation as an alternative cardiac fuel [47]. SGLT2is across trial programs consistently show improved renal outcomes and freedom from atrial arrhythmia [48,49].

##### Finerenone-Nonsteroidal Mineralocorticoid Receptor Antagonist (MRA)

Finerenone is a highly selective, potent nonsteroidal MRA that has received FDA approval for HFpEF in early 2026 following the landmark FINEARTS-HF trial. It showed a statistically significant reduction in worsening HF and related hospitalizations. Compared to steroidal MRAs such as spironolactone, finerenone portrays anti-inflammatory and antifibrotic effects by blocking both rapid non-genomic and slower genomic MR signaling in cardiovascular tissue, with a significantly lower risk of off-target hormonal side effects and lower incidence of hyperkalemia with better renal tolerability. The benefit observed in FINEARTS-HF was maintained among patients already receiving SGLT2i, supporting the use of finerenone as a complementary foundational therapy for HFpEF where applicable. [50,51].

However, in the FINEARTS-HF trial, while finerenone diminished the composite of total worsening heart failure events and cardiovascular mortality in patients with LVEF ≥ 40%, primarily by decreasing the incidence of worsening heart failure events, cardiovascular death alone was not significantly reduced. On the contrary, finerenone elevated the risk of hyperkalemia and necessitated monitoring of potassium levels and renal function.

#### 12.1.2. Pharmacological Interventions for Managing Underlying Comorbidities in HFpEF

Therapies such as loop diuretics, anti-hypertensives, and GLP-1 agonists, while not the main course of treatment for HFpEF, help in managing the underlying comorbidities, alleviating the hospitalization burden in HFpEF patients by offering symptomatic relief.

##### Hypertensive Interventions in HFpEF

The angiotensin receptor-neprilysin inhibitors (ARNIs) such as sacubitril/valsartan (Entresto) can be beneficial when used complementarily with other approved HFpEF interventions to manage blood pressure and can help counter cardiac remodeling. ARNIs were approved for HFpEF based on PARAGON-HF trial results where l sacubitril/valsartan reduced risk in patients with LVEF of 45% to 57%. The angiotensin II receptor blocker (ARB), while being standard in HFrEF, is not approved for HFpEF; however, it is still sometimes used to manage underlying conditions in HFpEF patients such as high blood pressure and kidney disease [52,53].

##### GLP-1 and GIP/GLP-1 Receptor Agonists (Incretin Therapies)

The emerging incretin-based agents have proven particularly effective in the obesity-cardiometabolic phenotype of the disease [54]. The STEP-HFpEF program with semaglutide demonstrated meaningful improvements in KCCQ-CSS scores, exercise capacity, and body weight reduction. However, confirmation of reduction in strictly categorized CV events is still pending. In the SUMMIT trial, the dual GIP/GLP-1 agonist tirzepatide showed a 38% reduction in worsening of HF events, with a consistent improvement in KCCQ-CSS, 6 min walk distance, and medication burden. The CMR sub-study of SUMMIT also showed reductions in left ventricular mass and pericardial fat tissue as cardiac mechanisms distinct from weight loss alone [55]. Beyond reducing plasma volume and hemodynamic load, these agents contribute to lowering the level of pro-inflammatory cytokines such as TNF-α, IL-1β, and IL-6 while increasing adiponectin, thereby exerting direct anti-inflammatory effects relevant to the metabolic HFpEF phenotype [54]. However, these findings support phenotype-specific therapy rather than general HFpEF treatment. Table 1 below details pharmacologic therapies used in HFpEF with their general mechanism of action, regulatory status, and phenotypic outline.

### 12.2. Investigational HFpEF Programs and Targets

This section discusses the HFpEF targets with preclinical translational benefits and those with proven translational potential and are being explored in early ongoing clinical trials. As various phenotypic axes and their mechanistic contributions are being uncovered in HFpEF, more explanatory targets are under investigation for therapeutic development.

#### 12.2.1. IL-6 Ligand Inhibition

Systemic IL-6 elevation is a consistent feature of inflammatory HFpEF phenotypes that drive STAT3-mediated endothelial dysfunction, fibroblast activation, and cardiomyocyte hypertrophy. Trials such as ATHENA and HERMES, ongoing Phase III clinical studies, evaluate the efficacy of ziltivekimab, a fully human monoclonal antibody that targets IL-6 ligand in patients with HFmrEF/HFpEF and systemic inflammation (elevated CRP level). ATHENA is focused on patient’s health-status and symptoms with KCCQ-CSS at 12 months, while HERMES evaluates clinical outcomes such as reduction in cardiovascular hospitalization and death.

In an anti-inflammatory trial “THE RESCUE” for CKD with high cardiovascular risk, ziltivekimab demonstrated proof-of-concept for strong cardiovascular biomarker lowering in cardiometabolic inflammatory disease [56]. While targeting inflammation is still a plausible therapeutic modality in HFpEF, it remains under clinical investigation and has yet to prove efficacy in patients, and results are expected in the next 4 years.

#### 12.2.2. NLRP3 Inhibition

The NLRP3 inflammasome through IL-1β and IL-18 secretion promotes fibrosis and tissue stiffness, contributing to HFpEF pathophysiology. While broad-spectrum anti-inflammatory drugs can have adverse side effects, preclinical studies involving selective NLRP3 inhibitors (MCC950, dapansutrile/OLT1177) showed reduction in left ventricular hypertrophy, fibrosis, and diastolic dysfunction in mouse HFpEF models. Anakinra, an IL-1 receptor antagonist, exhibited lowered CRP levels and improved exercise tolerance in HFpEF patients in D-HART phase I trials; however, it yielded mixed results in the D-HART2 phase II trial, providing benefit to patients in a higher EF class (EF ≥ 60%) [57]. Clinical development of oral NLRP3 inhibitors for HFpEF would be an important and mechanistically desirable frontier from existing therapies. Dapansutrile, an oral NLRP3 inhibitor in an early-stage clinical evaluation, has shown a favorable safety profile in HFrEF patients; however, efficacy and therapeutic profile are yet to be determined in HFpEF patients. In preclinical HFpEF models, dapansutrile reduces atrial inflammation, downregulates profibrotic gene expression, and lowers the risk of atrial fibrillation [58]. Notably, despite a convincing therapeutic promise of anti-inflammatory drugs, this axis remains under investigation for development of HFpEF therapeutics.

#### 12.2.3. Quest of NO–sGC–cGMP–PKG Signaling Restoration

Multiple pharmacological attempts are being made to restore the impaired NO–sGC–cGMP–PKG pathway that is at the mechanistic core of HFpEF pathophysiology. Compounds stimulating the soluble guanylate cyclase (sGC) enzyme to increase cGMP levels and relax blood vessels, such as BAY 41-8543 and vericiguat, were found to reduce fibrosis, improve endothelial function and attenuate cardiac remodeling in preclinical HFpEF models without altering NO levels. While vericiguat received approval for HFrEF, it did not help HFpEF patients, and BAY 41-8543 did not make it to human use due to its poor pharmacokinetics [59,60,61].

In CARDINAL-HF trials for HFrEF, PDE9 inhibition, which acts preferentially on natriuretic peptide-generated cGMP rather than NO-derived cGMP, was successfully used to increase cGMP plasma levels, providing clinical proof-of-concept for PDE9 inhibition efficacy in HF [62]. Experimental evidence suggests that oxidized PDE9 dramatically enhances cardiomyocyte passive stiffness in HFpEF through lower PKG activity, indicating PDE9 oxidation as a unique HFpEF-specific mechanism. Cycle-2-PEF is evaluating CRD-750, the PDE9 inhibitor that represents a new mechanism to enhance natriuretic peptide signaling; however, more focused research studies are needed to prove any definitive therapeutic potential [63,64]. While restoring NO-sGC-cGMP-PKG signaling is a desirable frontier in addressing HFpEF, putative pharmacological targets in this domain failed to verify therapeutic superiority and remain strictly exploratory.

#### 12.2.4. Aldosterone Synthase Inhibition

Vicadrostat (BI 690517) is under investigation in patients with HFpEF. It selectively inhibits CYP11B2, an aldosterone synthase, limiting the overproduction of aldosterone, which is known to worsen chronic heart failure and cause renal issues. It is being clinically evaluated for HFpEF in the EASi-HF trial to be used complementary to the SGLT2 inhibitors. In synergistic pairing with an SGLT2 inhibitor, Vicadrostat is hypothesized to enhance the therapeutic benefits and offers potential regulation of the potassium-level fluctuations often associated with aldosterone inhibition [65].

#### 12.2.5. Titin and Myocardial Passive Stiffness

Titin, an intra-sarcomere protein, determines cardiomyocyte passive tension in HFpEF through two molecular mechanisms: first, by isoform switching toward the less compliant N2B titin isoform (reduced N2BA/N2B ratio), which is regulated by the splicing factor RBM20. The other phenomenon is post-translational modification deficits, specifically, hypo-phosphorylation at PKA/PKG sites and hyperphosphorylation of the PEVK domain by PKCα. Pharmacologically, RBM20 inhibition improves diastolic function in HFpEF models. Metformin has been associated with favorable effects on titin compliance in experimental models, although its relevance as a direct HFpEF therapy remains uncertain [29].

#### 12.2.6. Cardiac Fibrosis Targets

Myocardial fibrosis represents a mechanistically relevant disease process and a potential source of imaging and a circulating biomarker in HFpEF. TGF-β/ALK5, galectin-3, collagen crosslinking via lysyl oxidase (LOX), and TIMP/MMP imbalances are well-validated drivers of cardiac fibrotic remodeling. Class IIa HDAC inhibitors reverse pathological histone acetylation programs and attenuate diastolic dysfunction in the murine HFpEF models [40,66]. BET/BRD4 bromodomain inhibitors suppress pro-fibrotic and pro-inflammatory gene expression by displacing BRD4 from super-enhancers, representing a broad epigenetic antifibrotic strategy, but efficacy against HFpEF must be proven in the clinic. The PIROUETTE, a randomized phase II trial with pirfenidone, which is an antifibrotic agent approved for treating idiopathic pulmonary fibrosis, evaluated its efficacy in HFpEF and found reduction in myocardial collagen content. While it provides a potential proof-of-concept for antifibrotic pharmacotherapy, the support comes through a modest imaging signal, and thus, larger-outcome trials are needed to confirm these findings [67].

#### 12.2.7. Cellular Senescence and PANoptosis

HFpEF, specifically in the aging phenotype, is strongly correlated with the accumulation of senescent cells and their pro-fibrotic and proinflammatory secretory cocktail called senescence-associated secretory phenotype (SASP). In ZSF1-obese rats, senescent immune and endothelial cells appear in peripheral blood, and increased circulating senescent leukocytes correlate with markers of cardiac and endothelial damage [68]. Senolytics such as navitoclax (ABT-263), a BCL-2/BCL-xL inhibitor, can selectively induce apoptosis in senescent cells and were found to reduce systemic inflammation, attenuate cardiac fibrosis with improved microvascular density, decrease BNP, enhance renal function, and alleviate pulmonary edema in the ZSF1-obese HFpEF model [69]. While the senolytics are under exploration in preclinical models, bridging the clinical gap presents safety and efficacy challenges [70].

PANoptosis is a recently defined, unique form of programmed cell death that integrates components of pyroptosis, apoptosis, and necroptosis. In the failing heart, excessive PANoptosis is driven by inflammatory triggers and leads to massive cardiomyocyte loss, arrythmias, and remodeling. Since PANoptosis can regulate inflammation, exploratory attempts are underway to evaluate the effect of its inhibition on the reduction of the severe cell loss and ventricular remodeling seen in heart failure. At the molecular level, pathways such as AMPK/mTORC1/autophagy, detyrosinated α-tubulin, and ZBP1-mediated PANoptosis have been implicated as novel therapeutic targets, and interventions targeting these pathways have demonstrated efficacy in preclinical models [71,72,73]. The Table 2 groups targets under investigation for advancing HFpEF therapeutics.

### 12.3. Alternative Mechanical Interventions

Nonpharmacologic approaches under investigation include stimulation-based technologies, nerve ablation therapy, and mechanical circulatory support devices, though their efficacy requires further research. Cardiac contractility modulation (CCM) has also shown encouraging signals in small studies but remains investigational in HFpEF and requires larger randomized evaluation [84]. Exercise training and cardiac rehabilitation are recommended to enhance exercise capacity, optimize medication therapy, and promote a healthy lifestyle, with specific targets including improved VO2 peak, medication adherence, risk factor modification, and psychosocial support.

Diagnostic algorithms such as the H2FPEF and HFA-PEFF scores target patient demographics, clinical characteristics, and echocardiographic parameters to identify HFpEF and guide management. Traditional heart failure therapies such as beta-blockers, angiotensin receptor antagonists, and angiotensin-converting enzyme inhibitors have been used, but their unequivocal benefit in HFpEF has not been proven, which calls for continued developmental need for novel targets and strategies [4].

### 12.4. Advances in HFpEF Diagnostic Methods–Biomarker and Precision Phenotyping

Biomarker research in HFpEF has identified several candidates for diagnosis and prognosis of the disease. While natriuretic peptides, including BNP and NT-proBNP, remain reliable biomarkers, their diagnostic accuracy is limited by the heterogeneity of HFpEF [85]. SERPINA3 has appeared as a promising candidate biomarker with high diagnostic value though calls for external validation, and galectin-3 independently predicts all-cause mortality and heart failure rehospitalization though the utility is context dependent and closely tied to renal function and longitudinal changes. Growth differentiation factor-15 (GDF-15) and soluble suppression of tumorigenicity 2 (sST2) are also under investigation, with sST2 serving as a biomarker of fibrosis and inflammation [86].

Inflammatory biomarkers such as C-reactive protein (CRP) and interleukin-6 (IL-6) are elevated in HFpEF and are associated with disease progression and outcomes [87]. Biomarkers reflecting myocardial fibrosis, such as high carboxy-terminal propeptide of procollagen type-I (PICP) and low carboxy-terminal telopeptide of collagen type-I to matrix metalloproteinase-1 (CITP:MMP-1) ratio, are associated with severe diastolic dysfunction and poor outcomes. Other novel biomarkers include FGF21 and TMAO, which may serve as diagnostic markers for HFpEF and renal function [88].

Multi-marker panels and machine learning approaches are increasingly used to enhance diagnostic and prognostic accuracy in HFpEF [89,90]. MicroRNAs and non-coding RNAs are also being explored as potential biomarkers, with some panels demonstrating the ability to distinguish HFpEF from HFrEF, though there is no consensus on their use due to methodological variability [33].

Although natriuretic peptides are crucial for HF diagnosis and risk classification, BNP and NT-proBNP levels should be evaluated clinically due to factors such as obesity, atrial fibrillation, renal impairment, and age. Biomarkers such as galectin-3, sST2, GDF-15, miRNAs, SERPINA3, FGF21, and TMAO can give insight into fibrosis, inflammation, metabolic stress, and vascular dysfunction. However, they should be used as adjunctive or investigative markers rather than standalone diagnostic tools. For clinical use, repeatable tests, defined thresholds, external validation across HFpEF symptoms, and proof of improved outcomes through biomarker-guided therapy are necessary.

In conclusion, conventional monotherapy approaches targeting isolated pathways have mostly failed to yield therapeutic breakthroughs in HFpEF, likely because of its multifactorial pathogenesis. However, the SGLT2 inhibitors have shown a clear beneficial impact on clinical outcomes. This emphasizes the need to refine therapeutic strategies by aligning them with patient-specific disease drivers. The HFpEF target landscape should thus be considered in the context of distinct phenotypes such as metabolic modulation and weight loss for the obesity-related phenotype, aggressive diuresis, and afterload reduction in the cardiorenal phenotype, and so on. Despite advances, gaps remain in our understanding of HFpEF targets. The heterogeneity of HFpEF, the interplay between comorbidities and cardiac dysfunction, and the lack of highly specific biomarkers challenge both diagnosis and treatment. Future research should focus on refining molecular and cellular targets, validating novel biomarkers, and developing personalized therapeutic strategies that address the systemic nature of HFpEF.

## 13. Experimental Models of HFpEF

Another aspect that has hampered HFpEF research progress is a lack of robust experimental models that recapitulate the syndrome’s full complexity, such as incorporating multiple comorbidities simultaneously and modeling functional and morphological manifestations of the disease. HFpEF models, ideally, should reflect the hallmark features of human HFpEF, i.e., diastolic dysfunction with preserved LVEF, elevated filling pressures, evidence of left ventricular concentric remodeling or hypertrophy, exercise intolerance, and multisystem involvement. However, it has been a challenge to capture the HFpEF heterogeneity and complex nature in a single animal model [2].

A range of in vivo models using small and large animals has been developed to capture specific HFpEF risk factors and phenotypes. In parallel, the in vitro systems, from simple monolayer cell cultures to complex organ-on-chip platforms, have been used to dissect cellular mechanisms and identify therapeutic targets. However, these models still struggle to reproduce the integrated, organ-level physiology of HFpEF. This section reviews the key in vivo and in vitro models, highlighting their contributions and translational limitations [2,91,92].

### 13.1. Animal Models in HFpEF

Animal models remain essential for replicating the complex, multisystem nature of the HFpEF and for validating findings from in vitro studies. Animal models, such as obese ZSF1 rats, high-fat diet and L-NAME-induced mice, and Akita diabetic mice, are widely used to mimic the metabolic, hypertensive, and diabetic components of HFpEF [93,94]. The “two-hit” protocol in mice, combining metabolic and hypertensive insults, rapidly induces HFpEF features and mortality, providing a robust model for mechanistic and therapeutic studies [95]. Large animal models, including porcine models with implantable soft robotic sleeves, offer exceptional versatility and tunability, closely recapitulating human hemodynamic phenotypes and addressing limitations of smaller animal models [96].

#### 13.1.1. Rodent Models

Small animals such as mice and rats are often used for mechanistic HFpEF research given their genetic tractability and cost-effectiveness but are limited by substantial physiologic differences among size and lifespans in modeling an aging-related, comorbidity-driven syndrome. Traditionally, single-hit rodent models simulate individual HFpEF risk factors. Chronic pressure overload triggered by a hypertensive environment induces left ventricular hypertrophy and diastolic dysfunction in rodents with hypertension-induced HFpEF. Spontaneously hypertensive rats (SHRs), while doing a great job of recapitulating HFpEF characteristic hallmarks at an early age, e.g., developing concentric left ventricular hypertrophy and impaired diastolic relaxation by 4–8 months of age with a near-normal EF, falter in advanced age and lean towards developing systolic failure. On the other hand, transverse aortic constriction (TAC) in mice leads to early diastolic abnormalities with preserved EF, but the simplistic overload approach does not feature the extracardiac irregularities correlated with human HFpEF, such as pulmonary congestion and multi-organ changes [92].

Leptin-deficient (ob/ob) or leptin receptor–mutant (db/db) mice demonstrate the metabolic and obesity axis in HFpEF recapitulating hyperglycemia and insulin resistance. As a result, these mouse models manifest systemic inflammation with left ventricular hypertrophy and diastolic dysfunction; however, in addition, they show reduced systolic function or hypotension, which is not a typical HFpEF feature. Similarly, Zucker diabetic fatty (ZDF) rats and obese rodent models induced with a “high fat diet” exhibit insulin resistance and moderate diastolic irregularities, but again, without overt heart failure signs in isolation.

Senescence-accelerated mouse models (e.g., SAMP8 strain) can mimic the effects of aging and show age-dependent diastolic dysfunction and interstitial fibrosis. While these models capture the aging-HFpEF phenotype, they mostly lack the specific comorbid triggers and do not fully mirror the human geriatric physiology. To better mimic the HFpEF multifactorial nature, multifactorial (multi-hit) rodent models were developed to combine comorbidities [97] The landmark HFD + L-NAME two-hit model combined metabolic stress (high-fat diet) with chronic N(omega)-nitro-L-arginine methyl ester (L-NAME) administration (NOS inhibition; hemodynamic, inflammatory hit) in young mice [98]. This two-hit model yielded mice with obesity, hypertension, glucose intolerance, left ventricular diastolic dysfunction, pulmonary congestion, and exercise intolerance—a profile resembling human HFpEF [15]. Mechanistically, this model revealed nitrosative stress as a primary HFpEF driver. These molecular findings were later confirmed in human HFpEF myocardial biopsy studies, showing higher translational validity for the nitrosative stress-related pathways [99]. However, the models are limited by two-stressor inducers and sub-strain variations, do not represent human hypertension, and do not capture contributions from other comorbidities such as the cardio–renal axis or adipose–cardiac crosstalk. To address some of the limitations, genetic variants have been generated, such as the HFD + renin AAV8 two-hit model, which incorporates RAAS-driven hypertension and exhibits 30–40% left ventricular hypertrophy, preserved systolic function, diastolic dysfunction, elevated natriuretic peptides, exercise intolerance, and transcriptomic dysregulations associated with human HFpEF molecular signatures [100]. While a great advanced experimental model, genetic modification limits pharmacological mechanistic purity.

DOCA-Salt and western diet models, along with Dahl salt-sensitive rat models, have also been successfully used to capture broken NO-cGMP-PKG signaling and neurohormonal contributions in HFpEF pathophysiology. ZSF1 obese, a hybrid of Zucker diabetic fatty and SHR/HF rats, constitutes a 3-hit model incorporating aging + HFD + mild renal impairment to induce HFpEF over many months. Notably, ZSF1 rats also exhibit sarcopenia and skeletal muscle dysfunction, paralleling the muscle weakness seen in human HFpEF, and have been used to dissect the senescence axis in HFpEF and test the senolytic therapies [2,69,101,102]. However, early mortality due to progressive renal failure limits longitudinal studies while complex genetics complicates mechanistic inference.

Nevertheless, the rodent HFpEF models face translational limitations. They often require extreme or non-physiologic interventions to induce HFpEF within the short lifespan of rodents. These conditions do not reflect gradual, long, and chronic human disease progression, nor the important aspects of human cardiac physiology (heart rate, calcium handling) and organ crosstalk. Thus, while rodent models are invaluable for mechanistic studies, researchers must complement them with additional approaches [92].

#### 13.1.2. Large Animal Models

Larger mammals such as dogs, pigs, sheep, and others more closely mimic human cardiac size, heart rate, and complex organ crosstalk dynamics, making them useful for translational HFpEF research. These models are especially important for testing devices related to non-pharmacological therapies and performing detailed hemodynamic monitoring [103]. However, these models present various critical challenges such as excessive cost, ethical concerns, difficulty in recapitulating full comorbidity clusters, and poor progression.

Chronic hypertension can be induced via surgical aortic banding or pharmacologic vasoconstrictors to generate pressure-overload models. For example, a porcine model of hypertensive HFpEF using ascending aortic constriction for several months exhibited left ventricular hypertrophy, diastolic dysfunction, and elevated left atrial pressure. These models recapitulate ventricular hypertrophy, concentric remodeling, and high filling pressures but require invasive procedures.

Diet-induced metabolic models can be generated using pigs or dogs fed with a high-calorie/high-fat diet to develop obesity, insulin resistance, dyslipidemia, and modest ventricular hypertrophy diastolic impairment. Combining diet with other factors, e.g., mineralocorticoid (DOCA) administration or nitric oxide synthase inhibition, can yield a more robust porcine HFpEF model, with hypertension and diastolic dysfunction after prolonged treatment. One group reported a triple-hit porcine model (hypertension + diabetes + hypercholesterolemia) with continuous ventricular hypertrophy pressure monitoring to mimic the human condition’s multi-factorial nature [104]. Given the female predominance in HFpEF, at least one model that used aged female Dahl salt-sensitive rats was explored for capturing gender-based differences. sex. However, typical large animal studies rarely extend long enough to simulate human aging [105].

Nevertheless, large animal models offer advantages of detailed hemodynamic and imaging evaluation, such as using human HFpEF diagnostic criteria like H2FPEF or HFA-PEFF scores to confirm the HFpEF-like state. They also provide a testing platform for device-based interventions, e.g., interatrial shunt devices and novel pacing strategies. However, current large animal models remain resource-intensive and typically focus on limited triggers, thus failing to fully model the disease complexity. Therefore, the field still needs to develop a widely accepted, high-fidelity animal model that fully captures complex HFpEF mechanistic processes, underscoring the urgency of developing novel experimental platforms [10,92].

### 13.2. In Vitro Models

In addition to organoid-based systems, the in vitro models have been pivotal in elucidating the role of hypertensive stimuli and peptide mimetics in cardiac hypertrophy, with studies demonstrating that FKBPL levels correlate with HFpEF progression [106]. Human cardiomyocyte models, including those derived from iPSCs, offer advantages over animal models by overcoming species differences and enabling the study of human-specific molecular pathways. Engineered heart tissue (EHT) systems, which assess contractility in hydrogel-based constructs, further facilitate the examination of disease effects on myocardial function in both HFpEF and HFrEF [107].

Molecular pathway studies using the in vitro models have illuminated the involvement of WNT signaling, the cGMP-PKG axis, and titin phosphorylation in HFpEF pathogenesis. For instance, porcupine inhibition has been shown to attenuate hypertrophy and fibrosis, while enhancing cGMP-PKG signaling prevents diastolic stiffness [108]. The in vitro administration of protein kinase A (PKA) has been shown to decrease passive force in human cardiomyocytes by increasing titin phosphorylation, a deficit observed in HFpEF and correctable by PKA or PKG [109]. The anti-inflammatory and antioxidative effects of empagliflozin have also been validated in human myocardium and murine models, supporting its therapeutic potential [11].

Non-coding RNAs, including microRNAs, long non-coding RNAs, and circular RNAs, have been investigated in vitro for their roles in microvascular dysfunction, inflammation, and fibrosis, further expanding the repertoire of molecular targets in HFpEF [33]. The in vitro models have also clarified the role of galectin-3 in diastolic dysfunction, with serum levels correlating with echocardiographic parameters in advanced HFpEF [110]. Additional studies using mouse cardiac fibroblasts have revealed that iNOS overexpression increases proliferation, migration, and collagen expression, effects that are reversible with specific inhibitors [111].

Primary cells, including cardiomyocytes, fibroblasts, and endothelial cells, are isolated from animal HFpEF models or from cardiac biopsies and are frequently utilized to understand heart failure mechanics. Calcium handling defects and titin phosphorylation irregularities have been found to correlate with diastolic dysfunction in cardiomyocytes in the in vitro HFpEF models [112,113]. Cardiac fibroblasts are incorporated in co-culture models with cardiomyocytes to understand fibrotic processes under pathological insults related to HF [28]. The ex vivo analysis of human HFpEF myocardium has revealed distinct gene expression patterns exhibiting high profibrotic and inflammatory gene signatures in comparison to HFrEF. However, isolated cellular studies cannot capture higher-order organ function or interplay with extracardiac systems that characterize HFpEF [112,114,115].

Human induced pluripotent stem cell (hiPSC)-derived cardiomyocyte (CM) models are marked as breakthroughs and allow investigation of cell-intrinsic differences between HFpEF and HFrEF or controls. For instance, hiPSC-CMs have been used to study inflammation-related molecular signatures and metabolic and contractile differences under conditions leading to development of HFpEF [26,116,117,118]. However, a major caveat in these studies is the immature, fetal-like physiology of the cells that limits direct extrapolation to adult HFpEF. This drawback prompts interest in advanced differentiation and tissue engineering techniques.

Engineered heart tissues (EHTs) and organoids, or 3D cardiac microtissues, derived from iPSCs, provide more physiologically mature and complex models. These constructs can incorporate multiple cell types and extracellular matrix, capturing aspects of myocardial structure and cell–cell interactions. Additional approaches such as mechanical loading and electrical stimulation can further promote cardiomyocyte maturation, yielding adult-like contractility and electrophysiological properties. Recent work demonstrated a “comorbidity-inspired” human cardiac organoid (hCo) model of HFpEF using iPSC-derived cardiac organoids. A multi-hit approach was taken, and the organoids were exposed to a combination of obesity, metabolic, and hypertensive insults to model HFpEF. The multifactorial comorbidity-based exposure led to enhanced cardiomyocyte hypertrophy, interstitial fibrosis, diastolic dysfunction, imbalanced energetics, and metabolic impairments, effectively recapitulating key features of human HFpEF in vitro [119]. While such models are highly valuable for dissecting HFpEF mechanisms in a human cellular context and can be utilized for high-throughput drug testing, they continue to lack the neurohormonal and mechanical circulatory complexity of a living organism.

Microphysiological systems (heart-on-chip) combine tissue engineering with microfluidic devices and allow precise control of mechanical stretch, electrical pacing, and the biochemical environment for cardiac tissues. These systems can simulate hemodynamic loading conditions while allowing real-time functional measurements. For example, microfluidic endothelial–cardiomyocyte co-culture platforms can model endothelial–myocyte crosstalk, demonstrating how endothelial inflammation impacts cardiomyocyte relaxation [120]. Heart-on-chip technology has also been utilized recently to demonstrate translational utility for gene and mRNA therapy development for cardiac diseases [121]. Cardiac organoids, by utilizing high-throughput platforms, can screen hundreds of compounds for their therapeutic potential and can easily generate functional data [122]. These technologies can be further developed and utilized as HFpEF experimental models for incorporating controlled and advanced multi-dimensional pathological insults and predicting therapeutic outcomes; however, lack of vascularization, batch-to-batch variability, and limited progression modeling remain big challenges.

Another exciting avenue originates in the convergence of cardiac organoid and EHT technologies with modern high-throughput multi-omics techniques. These platforms are enabling deep mechanistic interrogation of HFpEF that is impossible in animal models or clinical biopsy studies. The multi-omics integration in HFpEF models now encompasses bulk and single-cell RNA-seq, spatial transcriptomics, mass spectrometry-based proteomics, metabolomics, and lipidomics [123]. These advanced techniques are phenomenal in expanding molecular insights and undercover the underlying pathway biology, which generates additional paths to developing future precision therapeutics. For example, analysis of human HFpEF myocardium integrated gene expression, proteomic, and metabolomic data to reveal a distinct “cardiometabolic HFpEF” molecular signature highlighting impaired fatty acid oxidation and upregulated inflammatory pathways [25].

Single-cell RNA sequencing (scRNA-seq) studies can identify and track cell-type-specific changes in HFpEF myocardial tissues [124]. Spatial transcriptomics and proteomics further allow mapping of regional differences within the heart, such as localized fibrosis or capillary rarefaction. By applying these approaches to advanced models such as EHTs, organoids, and microfluidic systems, the researchers can map and identify new drug targets at molecular-level resolution [9,125].

Despite these advancements, in vitro models face challenges such as the immature phenotype of stem cell-derived cardiomyocytes and the limited complexity of three-dimensional organoids, which, while incorporating multiple cell types, still fall short of recapitulating the full architecture and function of human myocardium [126]. Nonetheless, the advanced in vitro platforms have distinct strengths: they utilize human cells, enabling the study of human-specific molecular pathways without species discrepancies. They also allow high-throughput perturbations such as CRISPR editing and screening platforms for microRNA or drug libraries to identify novel HFpEF mediators. Thus, human in vitro models complement the in vivo models by enabling detailed molecular investigations under human-like conditions.

Advances in computational modeling and machine learning present exciting opportunities to complement HFpEF research methodologies, integrating multi-dimensional data in the in silico models. Machine learning-based photomapping is aided in identifying mechanistic phenomena related to the phenotypic manifestation of HFpEF but also in identifying the proteomic clusters associated with the respective phenotypes [9,125]. Multi-scale computational models of cardiovascular physiology are also being developed that can simulate HFpEF hemodynamics and energetics in silico to help with testing hypotheses before moving into the in vivo or in vitro models. Integrating ML with in vitro data, researchers can also predict how genetic or pharmacologic perturbations will affect HFpEF organoids or EHTs, guiding efficient experimental design [9,127].

## 14. Comparative Fidelity and Translational Value

Despite the utility of these models, no single in vivo system fully encompasses all hemodynamic and multisystem features of HFpEF, reflecting the syndrome’s heterogeneity and the challenge of modeling its complex pathophysiology [93]. Chronic in vivo models are particularly valuable for studying disease progression, neurohormonal responses, and multi-organ interactions but are limited by excessive costs, ethical considerations, and variable disease progression. Reviews and meta-analyses of over 500 experimental protocols demonstrate the necessity for models that replicate not only diastolic dysfunction but also the full spectrum of HFpEF comorbidities, including hypertension, diabetes, obesity, and aging [94]. Table 3 lists key characteristics of HFpEF experimental models.

Comparative analyses reveal that the in vitro models excel in dissecting cellular and molecular mechanisms, enabling high-throughput drug screening and mechanistic studies, while the in vivo models are indispensable for capturing systemic, neurohormonal, and long-term effects, as well as for validating device-based therapies [128]. In silico and ex vivo models could further complement these approaches by providing scalable, cost-efficient platforms for initial evaluation and device testing, though they lack the complexity of living systems.

Most animal models are limited by their reliance on single or dual risk factors, while human HFpEF is driven by a constellation of metabolic, inflammatory, and hemodynamic insults [92]. The development of multi-factorial models, incorporating combinations of hypertension, obesity, diabetes, and aging, is a promising direction for future research [94]. Additionally, integrating advanced computational models with in vitro and in vivo systems might enhance the fidelity and predictive power of experimental platforms in future, and would facilitate phenotype-guided therapeutic development [128].

To summarize, though considerable progress has been made in modeling HFpEF, substantial challenges remain. The in vitro models provide mechanistic insights and platforms for drug discovery but are limited by their inability to capture systemic complexity. Animal models are essential for studying disease progression and therapeutic efficacy, yet are constrained by cost, ethical considerations, and incomplete phenotypic fidelity. Future efforts should focus on developing integrated, multi-factorial models that better reflect the clinical heterogeneity of HFpEF and on leveraging advances in computational modeling to inform experimental design and therapeutic testing. These integrated approaches will be critical for advancing our understanding of HFpEF pathophysiology and for the development of effective, mechanism-based therapies.

## 15. Roadmap for Next-Generation HFpEF Research and Therapy

This review discusses the HFpEF targets and models, addressing significant gaps in the current understanding of this complex syndrome. The global impact of HFpEF is substantial, affecting an estimated 32 million individuals worldwide, with a rising prevalence expected to surpass that of heart failure with reduced ejection fraction (HFrEF) soon [129]. Despite this burden, diagnosing and managing HFpEF remains challenging due to its clinical heterogeneity and the absence of universally effective therapies. Considering these advances, we outline a forward-looking strategy for HFpEF, with an emphasis on tailored phenotype-guided therapies and innovative comprehensive model systems. Our work provides insights into the molecular and cellular processes in HFpEF to delineate the HFpEF target landscape and critically evaluate available in vitro and in vivo models. This approach is particularly relevant given the ongoing need for improved diagnostic and therapeutic strategies tailored to the diverse phenotypes of HFpEF.

A notable area of agreement with recent studies is the use of SGLT2 inhibitors as the first pharmacologic class to demonstrate clinical benefit in HFpEF, likely due to their pleiotropic effects on metabolic, inflammatory, and hemodynamic pathways [130]. Our work further highlights ongoing trials with GLP-1 receptor agonists and the potential of other agents targeting specific pathophysiological mechanisms [131]. However, the translation of novel biomarkers such as sST2, galectin-3, and multi-omics signatures into clinical practice remains limited by technical, economic, and validation challenges [123].

Contradictions with current knowledge are primarily observed in biomarker utility, where some studies report high diagnostic and prognostic value for novel markers, while others highlight their limited specificity and lack of clinical translation [42]. These discrepancies can be attributed to differences in patient populations, comorbidity profiles, and methodological approaches, underscoring the need for large-scale, multi-center validation studies [16].

This study underscores the importance of advancements required in multi-marker panels and machine learning approaches for improving diagnostic accuracy and risk stratification. Deeper phenotyping and endotype trials are needed to effectively stratify HFpEF patients according to the phenotypic manifestation of the diseases. In future, the routine use of approaches such as multidimensional profiling systems, clinical scoring, biomarker panels, imaging, and genomics to group HFpEF patients may facilitate identification of biologically relevant subgroups. Embracing systems biology and the integration of multiscale data in HFpEF to utilize circulating biomarker signatures, imaging metrics, and genotypes is valuable in discovering surrogate markers for HFpEF endotypes and treatment responses. Incorporating machine learning in future may facilitate identification of clinically relevant HFpEF subgroups and candidate biomarker signatures that may support development of predictive therapeutic-response models.

Most available models, particularly in rodents, lack the comorbidities and systemic features characteristic of human disease, while large animal models, though more physiologically relevant, are constrained by cost, ethical considerations, and technical challenges [132]. Our analysis supports the view that developing multi-factorial models, incorporating combinations of metabolic, hypertensive, and inflammatory insults, is essential for advancing preclinical research and therapeutic discovery [18,91]. Furthermore, the use of scalable human organoid or EHT models of HFpEF remains indispensable to test hypotheses and screen therapies in human cells. A major milestone in advancing the precision therapeutic approach would be creating patient-specific in vitro therapeutic screening platforms for predicting drug response.

In conclusion, HFpEF’s complexity has defied one-size-fits-all solutions. A comprehensive approach that recognizes HFpEF’s heterogeneity, employs advanced human-relevant models, and targets specific mechanistic pathways offer the best path forward to therapeutic breakthroughs. By systematically connecting phenotypes to mechanisms and therapy, the field can advance towards a guided precision framework for treatment of HFpEF, addressing a syndrome that continues to be the most significant unmet need in heart failure. The advances in multi-omics, machine learning, and tissue engineering are converging to provide unprecedented insights into HFpEF. With these tools, the next generation of HFpEF models and trials can be designed to finally deliver phenotype-guided therapeutic approaches and improve outcomes for this diverse patient population [9,112].

Identifying molecular and cellular targets, coupled with developing more representative experimental models, will facilitate the discovery and validation of mechanism-based therapies. Integrating multi-omics data and machine learning holds promise for refining HFpEF phenotyping and personalizing treatment strategies [123]. Our work reinforces the urgent need for collaborative efforts between basic scientists, clinicians, and industry to translate these advances into improved outcomes for patients with HFpEF.

## 16. Conclusions

Heart failure with preserved ejection fraction (HFpEF) remains a highly heterogeneous syndrome driven by complex interactions among metabolic, inflammatory, vascular, and cardiac remodeling pathways. This inherent heterogeneity contributes to variable clinical presentations and differences in therapeutic responses, highlighting the need for a deeper mechanistic understanding of disease subtypes and patient phenotypes.

Our findings underscore that while progress has been made in identifying molecular pathways associated with HFpEF, currently available in vitro and in vivo models do not fully recapitulate the multimorbid nature of human HFpEF, which commonly involves obesity, diabetes, hypertension, CKD, and aging. This inadequacy continues to hinder the translation of preclinical discoveries into clinical practice. The application of innovative experimental models and advanced computational tools holds considerable promise, their utility still faces limitations due to inherent differences between experimental models and human disease, as well as challenges in validating novel biomarkers and therapeutic targets across diverse patient populations. Given the complexity of HFpEF pathophysiology and the heterogeneity of clinical presentations, it is evident that current therapies do not offer phenotype-sensitive disease treatment, reinforcing the importance of improved disease stratification in both preclinical and clinical studies.

Therefore, future research directions are essential to advance the field and overcome these challenges. These include focusing on developing more representative multi-factorial animal models that better capture the complexity and diversity of HFpEF. Furthermore, while precision medicine approaches, computational modeling, novel biomarkers, incorporation of patient-derived tissue and organoid systems are promising avenues, it is crucial to acknowledge that they remain investigational and require further validation before routine clinical implementation. Continued progress in these areas may ultimately facilitate more precise therapeutic development and improve outcomes for patients with HFpEF.

## Figures and Tables

**Table 1:** Mechanism of action (MOA) of pharmacologic therapies for HFpEF and underlying comorbidities.

Drug Names and Class	Primary Endpoint	Regulatory Status	HFpEF Phenotype	MOA	Current Clinical Use
SGLT2 Inhibitors					
Empagliflozin	CV-related hospitalization or worsening HF (first HHF or urgent HF visit); (EMPEROR-Preserved)	FDA-approved for HF regardless of EF	All HFpEF; benefits across LVEF ≥ 40%; regardless of diabetes status	SGLT2i inhibits sodium-glucose co-transporter 2 in proximal tubule → glycosuria, natriuresis, osmotic diuresis, reduced preload/afterload, improved cardiac metabolism and reduced inflammation/fibrosis.	First-line disease-modifying therapy in HFpEF (Class I for SGLT2i in HF)
Dapagliflozin	CV-related hospitalization, worsening HF (HHF/urgent HF visit); (DELIVER)	FDA-approved for HF regardless of EF.	All HFpEF + HFmrEF; diabetic and non-diabetic	SGLT2 inhibitor—same class mechanism as empagliflozin (natriuresis, osmotic diuresis, ketone body use, anti-inflammatory/anti-fibrotic effects)	First-line disease-modifying therapy; interchangeable with empagliflozin per guidelines; Class I recommendation
Non-steroidal MRA					
Finerenone	CV-related hospitalization or worsening + total HF events (HHF + urgent HF visits; first and recurrent); (FINEARTS-HF)	FDA-approved for HFmrEF and HFpEF (EF ≥ 40%)	HFmrEF/HFpEF (LVEF ≥ 40%); diabetic/non-diabetic, CKD patients	Non-steroidal mineralocorticoid receptor antagonist (ns-MRA)—selective MR blockade → reduced cardiac/renal fibrosis and inflammation with lower hyperkalemia risk vs. steroidal MRAs	Used as non-steroidal MRA in conjugation with SGLT2i, reduces the need for diuretics
GLP-1 Receptor Agonists for managing Obesity in HFpEF patients					
Semaglutide	KCCQ-CSS improvement + 6MWD change (co-primary, hierarchical). (STEP-HFpEF/STEP-HFpEF DM)	Not approved for HFpEF; approved for obesity (Wegovy)/T2DM (Ozempic).	Obese HFpEF (BMI ≥ 30); cardiometabolic HFpEF phenotype	GLP-1 receptor agonistincretin mimetic → weight loss, reduced visceral adiposity, anti-inflammatory effects, improved endothelial function, reduced insulin resistance, decreased epicardial fat Central satiety/weight regulation. improved hemodynamics via weight loss	The clinical trials show that use in obese HFpEF patients results in reduction of HF-related symptoms. Used for symptom relief and weight reduction; not yet guideline-recommended specifically for HFpEF; larger outcomes trial (SELECT-HF) ongoing
Tirzepatide	CV worsening HF events. (SUMMIT)	Not approved for HFpEF; approved for obesity (Zepbound)/T2DM (Mounjaro)	Obese HFpEF (BMI > 30); obesity-dominant phenotype	Dual GIP/GLP-1 receptor agonist—dual incretin action → greater weight loss than GLP-1 RA alone, improved insulin sensitivity, reduced inflammation, decreased adiposity; ↓ intramyocardial lipid; improved insulin sensitivity; systemic anti-inflammatory; ↓ filling pressures; improved left ventricular mass, ↓ hsCRP; ↓ NT-proBNP	Use for managing obesity and diabetes, SUMMIT trial showed reduction in worsening HF events; not yet in formal HFpEF guidelines [55]
Anti-hypertensive drugs					
Loop Diuretics (furosemide, bumetanide, torsemide)	No dedicated RCT with mortality/morbidity primary endpoint in HFpEF; used for symptom relief (congestion)	Not approved for treating disease, used for symptom relief in broad HF indications, prescribed for fluid overload and congestion management in HF, phenotypes with congestion benefit in general;	HFpEF phenotypes with congestion, volume overload	Potent natriuresis and diuresis → reduce intravascular volume, pulmonary/systemic congestion, and cardiac filling pressures.	provides symptomatic management; used in congested HFpEF patients; no mortality benefit proven but essential for quality of life and decongestion
ARNI (Sacubitril/Valsartan)	CV death or HF hospitalization: did not meet significance; (ARNI-PARAGON-HF) ARB-I-PRESERVE (irbesartan), CHARM-Preserved (candesartan): modest reduction in HF hospitalization	ARNI is FDA-approved for HFpEF/HFmrEF. Used in patients with EF in range 41–57%, female sex, and recent HF hospitalization; may benefit hypertensive/vascular phenotype. ARB: not FDA-approved for HFpEF, provides symptom relief as patients with hypertensive HFpEF may benefit	HFpEF phenotypes with hypertension	ARNI: Neprilysin inhibitor + ARB → increases natriuretic peptides (ANP, BNP) + blocks AT1 receptor → vasodilation, natriuresis, anti-fibrotic, anti-hypertrophic. ARB: AT1 receptor blockade → reduced aldosterone, vasodilation, anti-remodeling	ARNI: Used in select HFpEF patients (especially EF < 57%, women, hypertensive) ARB: Commonly used for blood pressure control and comorbidity management in HFpEF

(↓ = decrease, ↑ = increase).

**Table 2:** The summary of the emerging therapeutic targets that are under investigation for their therapeutic use in HFpEF patients.

Target	Category	Key Biology	Evidence Level	Translational Relevance	Notable Compounds	Limitations
Mitochondria dysfunction	Molecular target/metabolic pathway	Impaired energetics and oxidative stress [74]	Preclinical/Translational	Highly relevant to cardiometabolic HFpEF, skeletal-muscle dysfunction, exercise intolerance	Metabolic modulators; mitochondria-protective strategies	No validated HFpEF-targeted mitochondrial therapy; and comorbidity complexity limit translation
STING pathway (macrophage)	Molecular inflammatory target	Cytosolic DNA sensing, macrophage activation, inflammation [75]	Preclinical	Useful mechanistic link between sterile inflammation and myocardial remodeling	STING inhibitors; inflammasome adjacent strategies	No HFpEF clinical validation; immune suppression risk; target redundancy.
Clonal Hematopoiesis (CHIP)	Biomarker	Age-associated hematopoietic clones amplify inflammatory cardiovascular risk [76]	Translational/Observational	May help identify inflammatory HFpEF endotypes and trial-enrichment populations	JAK1/2 inhibitors, TET2-targeted strategies	Not yet actionable as routine HFpEF biomarker: causal vs. associative role remains incompletely defined.
Calcium cycling (SR)	Molecular target	Abnormal calcium reuptake and relaxation impair diastolic reserve [77,78]	Translational	Directly maps to impaired relaxation and exercise intolerance	SERCA2a gene therapy (MUSIC-HFpEF)	Arrhythmia and inotropy safety concerns; clinical benefit not proved; HFpEF heterogeneity dilutes signal.
RBM20 splicing	Molecular target	Controls N2BA/N2B titin ratio; RBM20 inhibition restores compliance [79]	Preclinical/mechanistic	Mechanistically attractive for stiffness-dominant HFpEF	Antisense oligonucleotides, small molecule RBM20 inhibitors	Safety risk if sarcomere splicing is altered broadly; no clinical HFpEF program validated
Splanchnic nerve ablation	Device/neuromodulation	Modulates venous capacitance and volume redistribution to reduce filling pressures [80,81]	Investigational/Early Clinical (REBALANCE-HF)	Potentially relevant for a volume-redistribution HFpEF subgroup	Catheter-based GSN ablation (Axon System)	REBALANCE-HF was safe/feasible but did not reduce exercise PCWP or improve outcomes in broad HFpEF; responder analyses remain exploratory
Interatrial shunting	Device	Left atrial decompression, exercise filling pressure reduction [82]	Investigational/Early Clinical Phase II (AFR-PRELIEVE) (Responder HF)	Mechanically relevant for elevated left atrial pressure without pulmonary vascular disease	Corvia atrial shunt; other atrial shunt platforms	REDUCE LAP-HF II showed no overall reduction in HF events or health-status improvement; possible responder subgroups require prospective validation
Cellular senolytic therapy	Molecular target	SASP clearance, anti-inflammatory, antifibrotic [69,83]	Preclinical/Translational	Relevant to geriatric HFpEF and multimorbid metabolic-inflammatory phenotypes	Dasatinib/quercetin-like strategies; senescence-pathway inhibitors	Human HFpEF outcomes absent; off-target toxicity, frailty, immune effects and dosing schedule uncertainty remain unresolved

**Table 3:** Key characteristics of HFpEF experimental models.

Model Type	Strengths	Limitations
Large Animal Models (Porcine)	Closer physiologic relevance, hemodynamic fidelity	Excessive cost, ethical constraints
Rodent Models	Recapitulate metabolic, hypertensive and diabetic factors	Species differences
Engineered Heart Tissue	Assess contractility, molecular pathway studies	Limited systemic interaction
Human Cardiac Organoids	Recapitulate fibrosis, oxidative stress, inflammation, high relevance	Immature phenotype, limited 3D complexity
Computational\In silico	Cost-effective, scalable, preliminary testing	Lack full biological complexity

## Data Availability

No new data were created or analyzed in this study. Data sharing is not applicable to this article.
